# Shaping the Next Generation: The Evolution and Impact of the European Association for Cardio-Thoracic Surgery Pre-Trainee Committee

**DOI:** 10.1093/icvts/ivaf245

**Published:** 2025-10-30

**Authors:** Samuel Burton, Amaya Ramirez, Nabil Hussein, İbrahim Gökçe, Anne Dijkstra, Vinci Naruka

**Affiliations:** Department of Surgery, Kingston Hospital, London, KT2 7QB, United Kingdom; Faculty of Medicine, University of Navarra, Navarra, 31008, Spain; Department of Paediatric Cardiac Surgery, Birmingham Children’s Hospital, Birmingham, B4 6NH, United Kingdom; Acibadem University School of Medicine, Istanbul, 34750, Turkey; Department of Cardio-Thoracic Surgery, Radboud University Medical Center, Nijmegen, 34750, The Netherlands; Department of Cardiac Surgery, St Bartholomew’s Hospital, London, EC1A 7BE, United Kingdom

## INTRODUCTION

International associations and leaders have long recognized the importance and value of actively engaging with surgical residents to support developing careers and the future of cardiothoracic surgery (CTS).^1^ With the global trend of formalizing CTS residency training programmes, the body of young professionals aspiring to such training needs to be acknowledged. The “Pre-Trainee” community primarily consists of medical students and doctors aiming to enter CTS residency. In modern medicine, Pre-Trainees are required to recognize the importance of networking, research, collaboration, and non-clinical skills development earlier in their careers. Over recent years, several international surgical associations have attempted to bridge the training pathway by facilitating Pre-Trainee engagement. This editorial will outline the rationale for and activities of one such association, the European Association for Cardio-Thoracic Surgery (EACTS) Pre-Trainee Committee.

## THE RATIONALE FOR PRE-TRAINEE ENGAGEMENT AND MEMBERSHIP

Numerous studies have demonstrated a lack of exposure and education to CTS throughout undergraduate medical education and early careers, with as few as 26% of students having any experience in the specialty.^2^ Due to its nature, there is concern that without adequate experience provided by medical schools and/or national/regional societies, CTS may lose talented applicants to alternative specialties. Additionally, there are Pre-Trainees with an existing interest in CTS who may not be able to access the skills development required for career progression, whether due to geographical access or limited resources. Such inaccessibility will result in less-experienced Pre-Trainees and, subsequently, less-experienced residents. Recognizing this educational gap, the EACTS Pre-Trainee Committee was established with support from the EACTS Council, as well as its Education and Residents Committees. The committee originated from the initiative of 2 Pre-Trainee members from different countries and 2 CTS residents involved with EACTS, specifically the Chair of the Residents Committee and a member of the Education Committee. The diversity of the group and the support of EACTS made it possible to launch the initiative.

As the committee’s activities evolved, it was decided to include 2 additional members. A selection process was conducted with the aim of recruiting medical students from countries not yet represented in the committee. The process involved a thorough evaluation based on specific criteria, focusing on leadership experience and commitment to CTS. Ultimately, 2 Pre-Trainee members from the Netherlands and Turkey were selected, with the goal of further enhancing the committee’s diversity in terms of nationality, gender, and perspectives. The Pre-Trainee Committee works very closely with the Residents Committee. When organizing the Next Generation webinars, topics are selected in collaboration with the Residents Committee to align with the Case Corner, ensuring that Pre-Trainee members can also participate in these webinars.

Acknowledging the differing financial restraints and educational needs of Pre-Trainees, EACTS extended their membership to include Pre-Trainees at reduced fees. This framework provides an equitable opportunity for all motivated Pre-Trainees to acquire and develop knowledge and competencies within the CTS discipline.

## THE NEXT GENERATION WEBINAR SERIES AND SURGICAL SKILLS DEVELOPMENT

With the aims of increasing Pre-Trainee interest and knowledge in CTS, the 2024 EACTS Next Generation Webinar Series was developed. A faculty of experienced CTS residents was invited to provide a 6-part webinar series, with each session moderated by a senior attending surgeon. The main goal was to introduce the world of CTS to medical students, offering insights into the specialty that would capture their interest at an appropriate level. For this reason, during the first season, an exhaustive selection of topics was made to ensure they were both engaging and useful for students in their academic exams.

The first sessions addressed CTS residency, with residents sharing their experiences and guiding Pre-Trainees on the pathway to the specialty. These interactive sessions gave pre-trainees an opportunity to ask questions directly to residents, something not always accessible at their own medical schools.

In the following webinars, we focused on providing Pre-Trainees with key concepts about the procedures most commonly performed in CTS. This approach aimed to ensure that, upon attending an operating room, pre-trainees would be better equipped to comprehend the procedures being performed. For this reason, when preparing the webinars, we focused on including many images and videos to make the presentations engaging. Sessions also employed clinical cases and test questions to encourage Pre-Trainees to participate and make the sessions interactive. In addition, the presentations were thoroughly reviewed to ensure they were adapted to the medical students’ level and could also be useful for studying for their exams. Most importantly, there was always time for questions, where Pre-Trainees could raise their doubts. During this part of the webinar, the great interest of the participants became evident, as there were always many high-level questions from them.

With the aim of providing a comprehensive program, the Pre-Trainee Committee has gone beyond technical surgical skills and has also focused on promoting research. In the second season of the Next Generation webinars, the first session was dedicated to research, a topic that is not strongly encouraged in most medical schools. This session attracted a large audience and addressed various aspects of research, providing guidance on how to begin engaging in research from the early stages of medical school. The webinar titled *Developing a Career in Cardiothoracic Research* clarified the pathway for many students.

Educational and interaction programmes in CTS have demonstrated to strengthen student engagement and interest.^3^^,^^4^ Cost of training has been shown to be an educational barrier during surgical residency.^5^ In response, the Next Generation Series was made free for members to promote education access to Pre-Trainees of all backgrounds. A diverse faculty from all areas of the specialty was selected to present the series, to address previously identified barriers to training, including representation and gender bias.^6^^,^^7^

Across the 2024 6-part series, a total of 420 individual session registrations were made by Pre-Trainees from 26 countries. To our knowledge, this is one of the highest international representations for Pre-trainees in CTS. Demographic analysis of the first session feedback demonstrated 43% of participants identify as women and 77% as medical students. Total series feedback analysis demonstrated 98% of participants felt the session met their expectations, with 96% scoring the quality of sessions as “Good’’ or “Excellent’’ on a 4-point scale. Overall, 98% of all attendants reported that they would recommend this series to a colleague or friend. Subsequently, the Next Generation Series was approved for a second 6-part series in 2025. Considering the feedback from the initial series, sessions on transplant and congenital surgery were included in the curriculum of Series Two, as well as topics on research, major aortic surgery, and valvular surgery.

To better understand Pre-Trainees’ motivations, concerns, and perceived barriers, we conduct surveys at the end of each webinar. These surveys collect demographic data, gather opinions on the activities, and invite suggestions for improvement. In addition, committee members remain continuously open to feedback and actively engage with Pre-Trainees during the Annual Meeting, making themselves available as points of reference for ideas and input. As the initiative is still in its early stages, we plan to expand these efforts with additional surveys to gain deeper insight into Pre-Trainees needs and to tailor support more effectively in the future.

Feedback analysis revealed a recurring gap in surgical skills in Pre-Trainees, expressing a desire for additional education focused on the technical and operative aspects in CTS. Subsequently, a new, extended surgical skills online course curriculum was developed to meet the identified requirement of Pre-Trainees. This facilitated students of all backgrounds to employ accessible, low-fidelity simulation-based skills in their training. The creation of the surgical skills webinar represented a significant challenge, as delivering the sessions remotely made it difficult to effectively incorporate the practical component into the webinar. First, a detailed program was developed outlining the skills that were essential for Pre-Trainees, such as: knot tying, suture types, instrument handling, suturing, purse string sutures and an introduction to coronary anastomoses. Second, instructional videos tailored for medical students were recorded by CTS residents, explaining step by step how each procedure should be performed. In addition, a video library was created so that Pre-Trainees could review the explanation of the procedures whenever needed. The creation of this library was considered fundamental, as there are currently very few educational videos on specific CTS procedures designed for medical students. In addition, participants were provided with a list of materials they could acquire to create a kit to perform the procedures during the webinar.

On the day of the surgical skills session, 2 CTS residents were present to explain the pre-recorded videos to the participants. The videos were shown sequentially, and after each video, time was allocated for Pre-Trainees to practice the skills using the kits they had acquired. During this time, they could ask questions and receive feedback from CTS residents. Finally, the videos of coronary anastomoses were shown, emphasizing to the participants that they were not expected to master the skills at this stage, but having access to the videos would be helpful for them in the future. At the end of the webinar, an additional half hour was provided for practice and to address questions.

Additionally, recognizing the value of high-fidelity simulation, the Pre-Trainee Committee have collaborated with the EACTS Learning Lab to produce a new tailored Pre-Trainee Valve Surgical Skills course for the 2025 EACTS Annual Meeting, using validated simulation-based learning.^8^

## THE EACTS ANNUAL MEETING AND EDUCATIONAL GRANTS

The EACTS Annual Meeting represents an unprecedented opportunity to foster Pre-Trainees’ surgical knowledge, build international collaborative relationships, and develop mentoring opportunities. EACTS has aimed to develop a new space for Pre-Trainees at the Annual Meeting and facilitate early career development through new Sessions and Francis-Fontan Education Grants. The 2024 Annual Meeting in Lisbon successfully hosted 8 Medical Student Francis-Fontan Grant recipients following a competitive 2-phase application cycle. Recipients were provided with free meeting registration and a travel/accommodation stipend, as well as an introduction to the meeting and networking support. Following the success of the first EACTS Annual Meeting Pre-Trainee session and Grant Programme, the Pre-Trainee Committee is facilitating the first Pre-Trainee Abstract Session and renewal of the Annual Meeting Grant Programme. The newly designed Abstract Session offers Pre-Trainees the opportunity to showcase their emerging research and to improve on essential academic skills within an international, peer-reviewed setting.

Recognizing the value of visiting electives/observerships for medical students, the 2025 Francis-Fontan Elective Grant was opened to award three students with a financial stipend to facilitate further experience in CTS. Such opportunities are invaluable in developing surgical skills and facilitating mentorship relationships, particularly crucial to academic and personal professional growth.^9^

Since the creation of the committee, we have engaged with pre-trainees to gather their suggestions and feedback. One of the most requested initiatives has been the establishment of a mentorship programme to allow pre-trainees to connect with senior surgeons. The development of this programme has recently begun and still requires a structured network. An independent committee has been created, comprising residents, senior surgeons, and members of the Education Committee, with the aim of establishing a network that offers students opportunities in terms of rotations and research.

## CONCLUSION

The EACTS Pre-Trainee Committee was established to engage with medical students and pre-training doctors, offering them exposure to CTS and supporting those interested in pursuing a career in the specialty. A range of educational and career advancement initiatives, such as the Next Generation Webinar Series, Surgical Skills Course and the Pre-Trainee Francis-Fontan Grants, were created to generate interest in the specialty and offer support for career development while promoting diversity and inclusion. The Pre-Trainee Committee has developed a new international collaborative space to support the next generation of cardio-thoracic surgeons, produce skilled future residents, and advance the field of CTS.

## Data Availability

The data underlying this article will be shared on reasonable request to the corresponding author.
